# TLBO-Based Adaptive Neurofuzzy Controller for Mobile Robot Navigation in a Strange Environment

**DOI:** 10.1155/2018/3145436

**Published:** 2018-03-05

**Authors:** Awatef Aouf, Lotfi Boussaid, Anis Sakly

**Affiliations:** ^1^Department of Electrical Engineering, National Engineering School of Sousse, University of Sousse, BP 264, Erriadh, 4023 Sousse, Tunisia; ^2^Laboratory E*μ*E, Faculty of Sciences of Monastir (FSM), University of Monastir, Av. Ibn El Jazzar Skanes, 5019 Monastir, Tunisia; ^3^Research Unit of Industrial Systems Study and Renewable Energy (ESIER), National Engineering School of Monastir (ENIM), University of Monastir, Av. Ibn El Jazzar Skanes, 5019 Monastir, Tunisia

## Abstract

This work investigates the possibility of using a novel evolutionary based technique as a solution for the navigation problem of a mobile robot in a strange environment which is based on Teaching-Learning-Based Optimization. TLBO is employed to train the parameters of ANFIS structure for optimal trajectory and minimum travelling time to reach the goal. The obtained results using the suggested algorithm are validated by comparison with different results from other intelligent algorithms such as particle swarm optimization (PSO), invasive weed optimization (IWO), and biogeography-based optimization (BBO). At the end, the quality of the obtained results extracted from simulations affirms TLBO-based ANFIS as an efficient alternative method for solving the navigation problem of the mobile robot.

## 1. Introduction

The use of mobile robots in many applications such as security, medicine, industry, space exploration, and many other fields is growing day by day. This autonomous agent must be able to navigate in the strange environment with the aim to accomplish these applications. Therefore, robot navigation is one of the essential problems in the robotics fields which can be categorized into local and global path planning.

In the global path planning, the environment is completely known to the robot. Various techniques have been suggested for global navigation, that is, Voronoi graph [1], potential field methods [2], grids [3], and visibility graph [4]. In the local path planning, the mobile robot is able to control its motion autonomously employing different sensors. Many intelligent methods are developed by many researches to solve the local navigation problems such as particle swarm optimization [5, 6], genetic algorithm [7, 8], ant colony optimization algorithm [9, 10], cuckoo algorithm [11, 12], simulated annealing algorithm [13, 14], invasive weed optimization [15], biogeography-based optimization [16], neural network [17, 18], and fuzzy logic [19, 20].

The complexity of the fuzzy logic system is found in the partition of the membership functions and the number of the rules. However, the complexity of the neural network systems is the selection of the optimal architecture and the synaptic weight. To overcome these problems, neurofuzzy models for robot navigation are developed [21, 22]. In [23], the authors have designed a neurofuzzy controller for mobile robot navigation in an unknown environment. They have employed the neural network to train the robot to arrive at the goal and the fuzzy system is used to control the velocities of the robot.

The adaptive neurofuzzy system combines the automatic adjusting of the fuzzy parameters and the adaptability of the neural networks. Robot navigation using adaptive neurofuzzy system has been developed by Pothal and Parhi [24]. The navigational controller receives data from sensors and gives the steering angle as an output. Simulations results are tested in different environments and proved that ANFIS controller is efficient.

Deshpande and Bhosale [25] have solved the navigation of a nonholonomic mobile robot using ANFIS controller. In [26], the authors have used ANFIS controller in a strange environment to avoid collision with obstacles. They have offered various simulations exercises using KiKS Simulator. Another method for path planning and avoiding obstacle has been addressed by Mohanty et al. [27]. It is the use of multiple adaptive neurofuzzy systems. The output of the navigational system is the velocities of the wheels. Experimental results prove the validity of the designed approach. In [28], Al-Mayyahi et al. have suggested an ANFIS controller for navigation of autonomous vehicle. They have developed four ANFIS controllers to command the angular velocity of the left and right wheels and the heading angle between the goal and the robot.

In [29], the authors developed a PSO-based neurofuzzy method in order to generate a collision-free path in an unknown environment. In [30], an IWO-based adaptive neurofuzzy controller for path planning is suggested. In this novel approach, the authors use the invasive weed optimization to tune the premise parameters of the ANFIS controller.

Two of the most important problems are the training and updating of the different parameters in the adaptive neurofuzzy inference system. The antecedent parameters of the fuzzy membership functions are usually determined by the gradient descent algorithm, but the calculation of the gradients is complicate and can lead to the local minimum. As a result, the precision can be affected.

To get over this problem, a method benefiting from the combination of ANFIS and Teaching-Learning-Based Optimization (TLBO) algorithm is suggested to solve the navigation task of the mobile robot. Different optimization algorithms demand different parameters that affect the response of the algorithm. Unlike these intelligent optimization techniques, TLBO does not demand any parameters to be adjusted.

Wu et al. have [31] presented a path planning problem based on an improved TLBO algorithm called Nonlinear Inertia Weighted Teaching-Learning-Based Optimization (NIWTLBO). This new approach has higher precision in searching for the optimal collision-free path.

In [32], the authors have proposed an Improved Teaching-Learning-Based Optimization (ITLBO) for an optimal trajectory for robotic manipulators.

A navigation problem approach based on TLBO was developed by Ansari and Katiyar [33] to calculate the shortest path from source to final destination without collision with obstacles.

In [34, 35], Savsani et al. have applied a Teaching-Learning-Based Optimization algorithm in order to optimize the trajectory for a 3R robotic arm. The results show the significance of TLBO over other intelligent optimization algorithms.

TLBO is introduced in this work in order to improve the performance of the ANFIS by training the parameters of the membership functions and thereafter reducing the root mean square error.

This paper is organized into six sections.  Section 2 introduces the kinematic modelling of the differential mobile robot.  Section 3 describes in brief four evolutionary algorithms. In Section 4, simulations results are discussed. A comparative study is carried out in Section 5.  Section 6 concludes and outlines the future of our work.

## 2. Khepera III Kinematic Model

In this work, we employed a differential mobile robot called Khepera III [36] to simulate the suggested navigational algorithm. Khepera III is equipped with nine infrared sensors used for distance measurements, two DC motors, and two encoders to give its real position.

**Figure 1:**
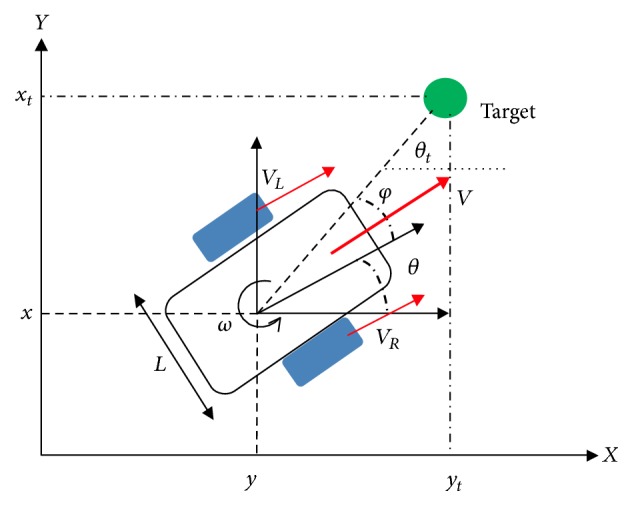
The kinematic model of Khepera III.

Figure 1 illustrates the position (*x*, *y*) and the orientation*θ* of the mobile robot in the Cartesian coordinate system. The desired target is represented by the coordinates (*xt*, *yt*).

The mathematical kinematic model is made through the link between the derivate of the position and orientation of the mobile robot and its linear (*v*) and angular (*ω*) velocities. It is given by these three equations:

(1) dxdt=vt×cos⁡θt,dydt=vt×sin⁡θt,dθdt=ωt.

## 3. Evolutionary Trained ANFIS Algorithms

### 3.1. Particle Swarm Optimization

The PSO technique is a swarm intelligence method member of a large category for solving the optimization problems. It is a population-based search algorithm, where each individual is referred to as particle and represents a candidate solution.

The notion of the PSO algorithm is that particles just move around multidimensional search space to approach the optima. Initially, a population is randomly created and set into movement. Each particle adjusts its position based on both its own experience and the neighboring particles' experience. At the end of each iteration, all particles value the fitness and move toward better positions. The velocity of each individual is a stochastic variable and can vary with respect to the distance from its best position. For the standard algorithm, the velocity *v*_*j*_^(*k*)^ and position *x*_*j*_^(*k*)^ of each particle in iteration *k* can be computed as follows:

(2) vjk+1=wvjk+c1r1pbest−xjk+c2r2gbest−xjk,xjk+1=xjk+vjk+1.

The parameters *c*_1_ and *c*_2_ set the relative pull of *p*_best_ and *g*_best_ and the parameters *r*_1_ and *r*_2_ which are uniformly distributed random variables in the range of [0, 1] help in stochastically varying these pulls.

### 3.2. Biogeography-Based Optimization (BBO)

Biogeography-based optimization is a novel evolutionary algorithm and metaheuristic, which is inspired by the biogeography concepts: speciation (the evolution of new species), the migration of species between islands, and the extinction of species. The algorithm was originally proposed by Simon in 2008 [37].

In biogeography-based optimization, every habitat is considered as an individual and every individual has its habitat suitability index (HSI) with the aim to evince its goodness. Habitat that has high HSI represents the good solution and habitat that has the low HSI represents the poor solution.

Through the process of immigration, a lot of novel features will be transmitted from high-HSI habitats to low-HSI habitats. Thus, emigration and immigration are two operators that are used to optimize a solution for the optimization problem.

### 3.3. Invasive Weed Optimization

The invasive weed optimization (IWO) is a nature-inspired metaheuristic algorithm. It was developed for the first time by Mehrabian and Lucas in 2006 [38]. The process of the IWO starts with initialization of a random population which is speared over the defined search space. Then, each seed produces flowering plants. These plants are ranked based on their fitness value before producing new seeds. In other words, the number of seeds varies linearly between the minimum seed production *S*_min_ and the maximum seed production *S*_max_. These seeds are randomly scattered over the search space by a normal distribution with mean equal to zero and varying standard deviation. The equation of the standard deviation for each generation is given as follows:

(3) $$\begin{array}{c} \sigma_{iter}={\left(\;\frac{{iter}_{max}-iter}{{iter}_{max}}\;\right)}^{n}\left(\;\sigma_{initial}-\sigma_{final}\;\right)+\sigma_{final}, \end{array}$$

where iter_max_ is the maximum number of iterations, *σ*_initial_ and *σ*_final_ are the initial and final deviation, respectively, and *n* is the nonlinear modulation index.

The novel generated seeds grow and produce plants. They are classified together with their parents on the basis of fitness values. The plants that have the lower value of fitness are remote to attain the maximum number of admissible plants in the colony *P*_max_.

### 3.4. Teaching-Learning-Based Optimization (TLBO)

Teaching-Learning-Based Optimization algorithm was proposed for the first time by Rao et al. in 2011 [39]. It is a metaheuristic algorithm inspired by process of teaching and learning through a simple mathematical model of knowledge amelioration gained by the students in the class [40].

Like other evolutionary algorithms, TLBO is also based on population method, which employs a population of solution to proceed for the search of the optimum solution. The population of solution is considered as a class of students.

In the optimization algorithm, the population of solutions contains many different design variables. In Teaching-Learning-Based Optimization, different variables correspond to different subjects given to students and student's result corresponds to the “fitness” function as in other optimization methods based on population. So far, teacher is the best obtained solution.

The working procedure of TLBO consists of two phases. The first part is the “Teacher Phase.” In this phase, students learn from teacher. The second part is the “Learner Phase.” In this phase, students learn via the interaction between learners.

## 4. Simulation Results

The principal purpose of this paper is to predict the optimized angular velocity for Khepera III using TLBO-based ANFIS controller.

The optimization of the different parameters of the fuzzy system is achieved when the error between the target and the actual output is minimized.

The most important step in implementing optimization algorithms is to choose the appropriate objective function. In this work, the objective function of all considered algorithms, PSO, IWO, BBO, and TLBO, is the root mean square error (RMSE). It is defined as follows:

(4) $$\begin{array}{c} RMSE=\sqrt{\frac{1}{k}\overset{K}{\underset{j=1}{\sum }}{\left(\theta_{a}-\theta_{p}\right)}^{2}}, \end{array}$$

where *θ*_*a*_ is the actual value of the steering angle and *θ*_*p*_ is the predicted value of the steering angle obtained from the PSO, IWO, BBO, and TLBO-based ANFIS controller and *k* is the number of observations.

For PSO, inertia weight (*w*) = 1, personal learning coefficient (*c*1) = 1, and global learning coefficient (*c*2) = 2; for IWO, minimum number of seeds (*S*_min_) = 0, maximum number of seeds (*S*_max_) = 5, sigma_initial (*σ*_initial_) = 0.5, sigma_final (*σ*_final_) = 0.01, and nonlinear modulation index (*n*) = 2; for BBO, mutation coefficient (*m*) = 0.1. TLBO algorithm does not demand any parameters for its running. This is one of the distinctive features of the TLBO compared with other intelligent algorithms. The chosen number of iterations, for the four considered algorithms, is 1000 and the size of population is 25.

Table 1 exhibits a sample of training and testing dataset that is employed in the learning process used by the ANFIS for go to goal and avoidance obstacle behavior.

**Figure 2:**
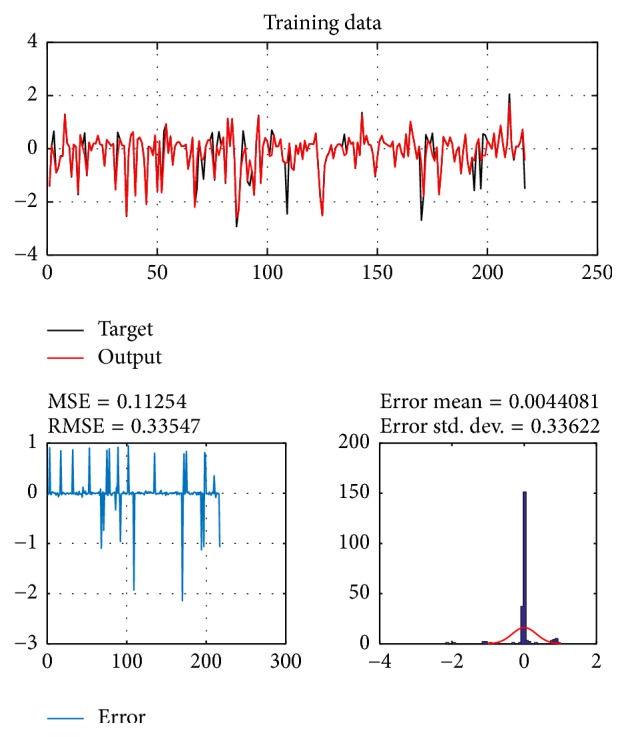
Training data of go to goal and avoidance obstacle behavior using PSO.

**Figure 3:**
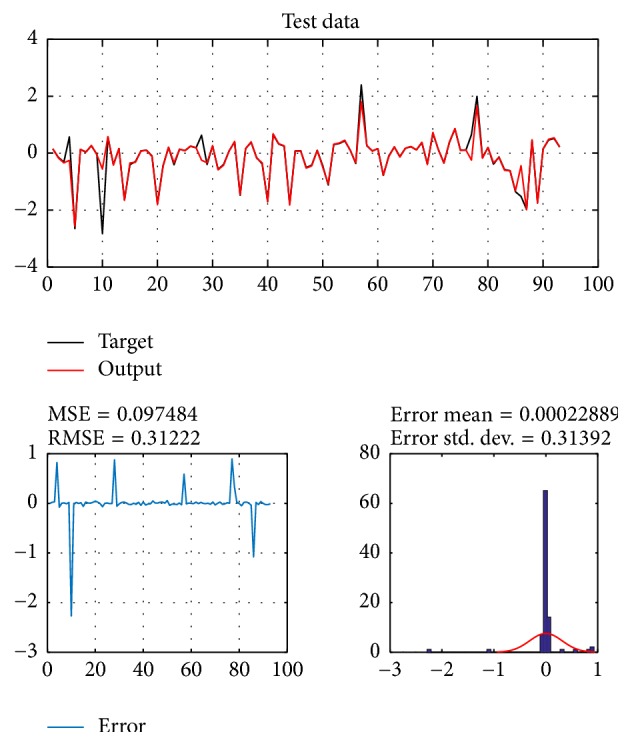
Testing data of go to goal and avoidance obstacle behavior using PSO.

**Figure 4:**
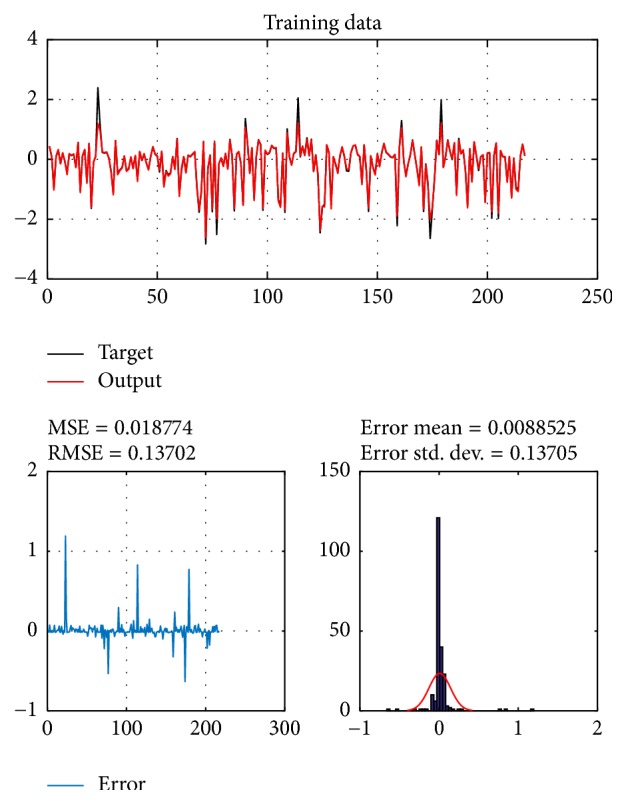
Training data of go to goal and avoidance obstacle behavior using IWO.

**Figure 5:**
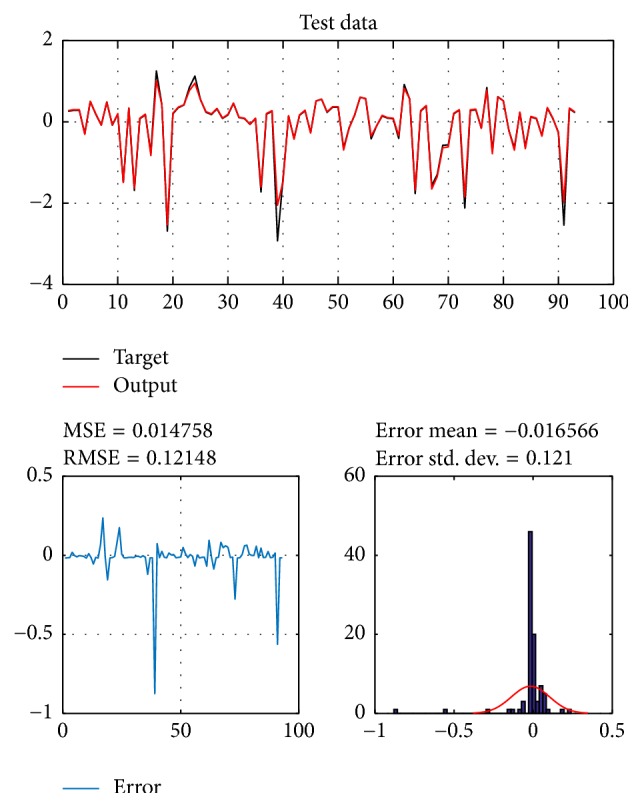
Testing data of go to goal and avoidance obstacle behavior using IWO.

**Figure 6:**
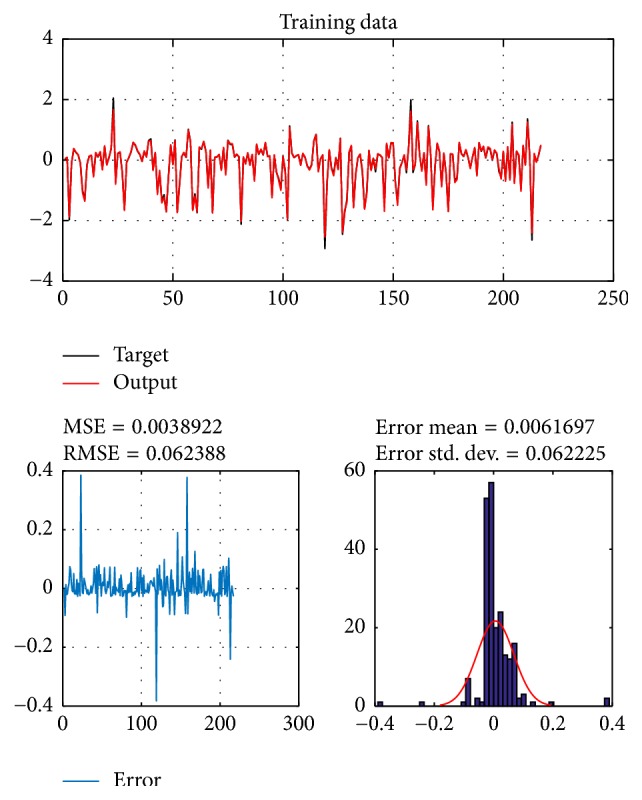
Training data of go to goal and avoidance obstacle behavior using BBO.

**Figure 7:**
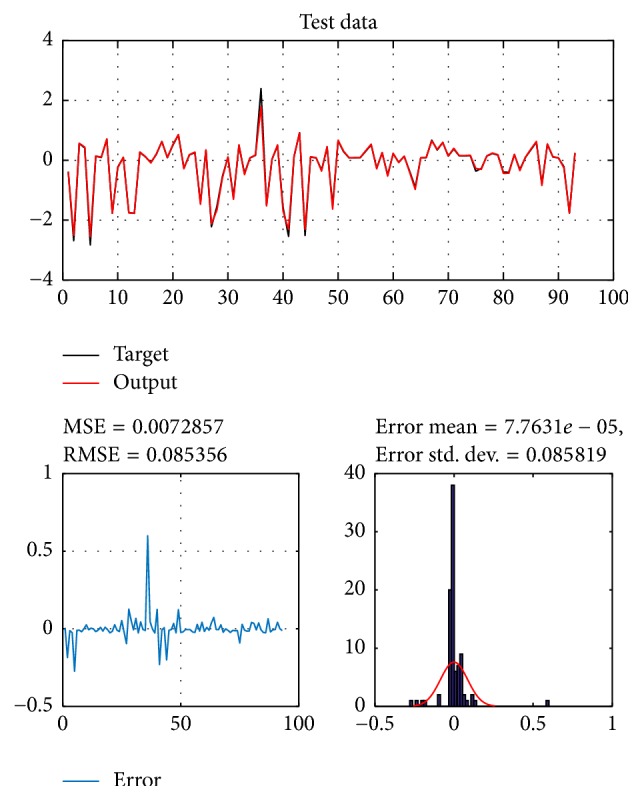
Testing data of go to goal and avoidance obstacle behavior using BBO.

**Figure 8:**
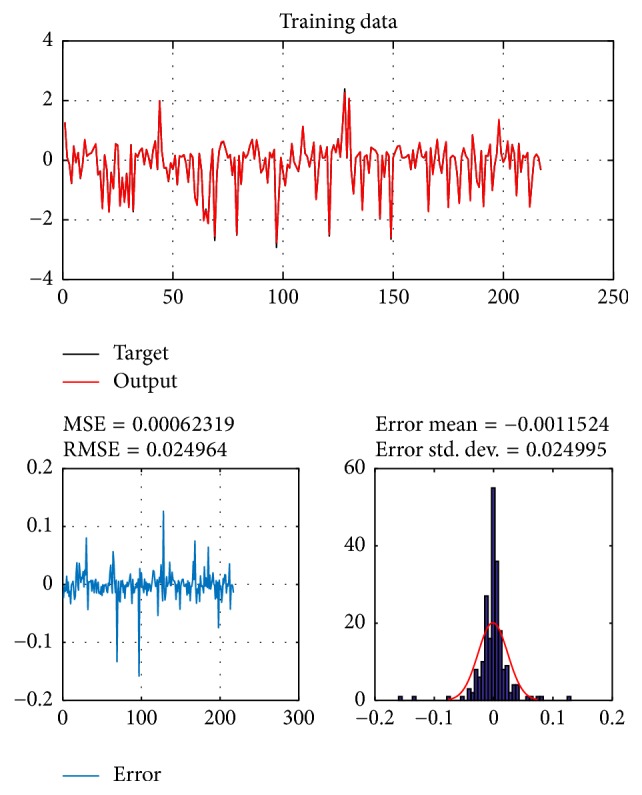
Training data of go to goal and avoidance obstacle behavior using TLBO.

**Figure 9:**
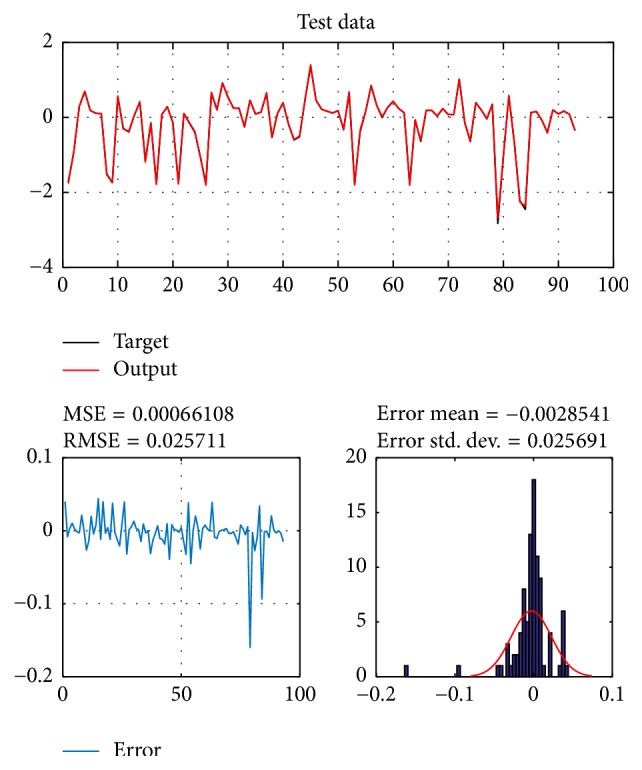
Testing data of go to goal and avoidance obstacle behavior using TLBO.

A database of 310 input-output pairs is prepared based on knowledge of Khepera III. 217 datasets are randomly chosen as training patterns and the remaining 93 datasets are employed as testing patterns to confirm the efficiency of the suggested ANFIS structure. Figures 2, 4, 6, and 8 depict the training results of the angular velocity using PSO, IWO, BBO, and TLBO algorithms, respectively. Figures 3, 5, 7, and 9 show the testing results of the angular velocity of the mobile robot employing the same four optimization algorithms.

The TLBO method gives the outputs with small errors.

MSE, RMSE, mean error, and std. dev. of the training and testing data using PSO, IWO, BBO, and TLBO algorithms are summarized in Table 2.

When they are compared with each other, it can be clearly observed that the best outcomes are obtained from the ANFIS trained with the Teaching-Learning-Based Optimization (TLBO) algorithm.

**Figure 10:**
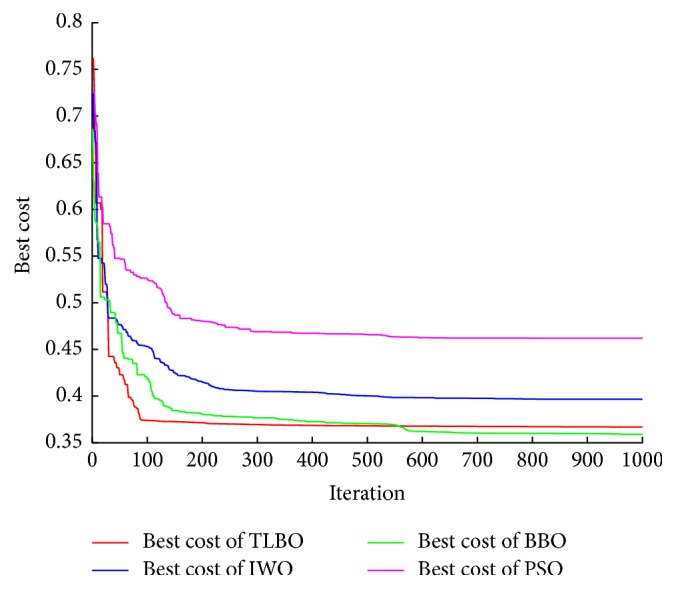
Convergence of the different optimization algorithms.

The convergence of the algorithm plays an essential role in the optimization algorithm. The convergence of best cost averaged for 15 independent runs of all considered algorithms is illustrated in Figure 10. It can be clearly observed that the TLBO algorithm has good convergence compared to other algorithms. It converges after almost 95 iterations, whereas BBO algorithm takes about 150 iterations, IWO algorithm takes about 250 iterations, and PSO algorithm takes about 300 iterations.

## 5. Comparative Study with Other Intelligent Algorithms

In this section, a comparative study has been made of the suggested evolutionary trained ANFIS controller (TLBO-based ANFIS) and the other intelligent navigational controllers using the PSO, IWO, and BBO algorithms in the graphical mode. We have presented simulations to prove the ability of the developed path planner to lead the navigation of the mobile robot in various situations. All simulations are performed using Matlab environment using sim.I.am simulator which is developed by GRITS Laboratory of Georgia Tech University.

**Figure 11:**
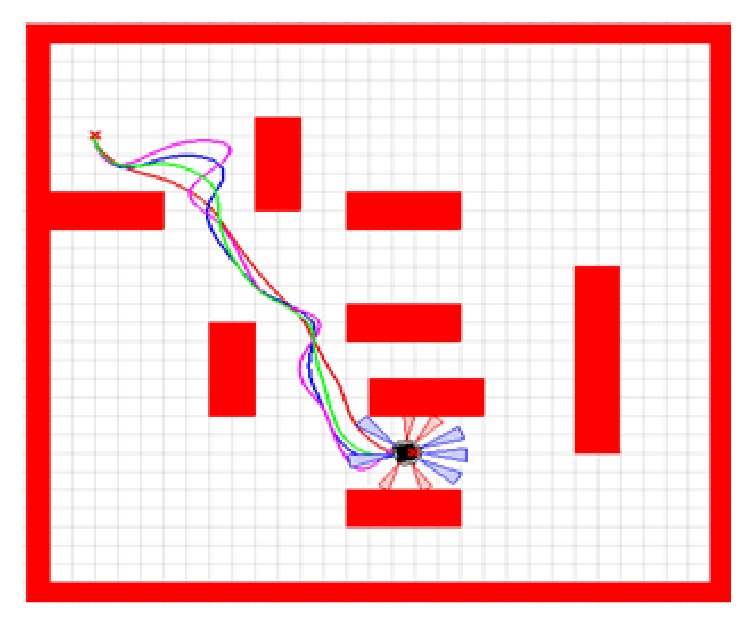
Motion of the mobile robot in the first strange environment.

**Figure 12:**
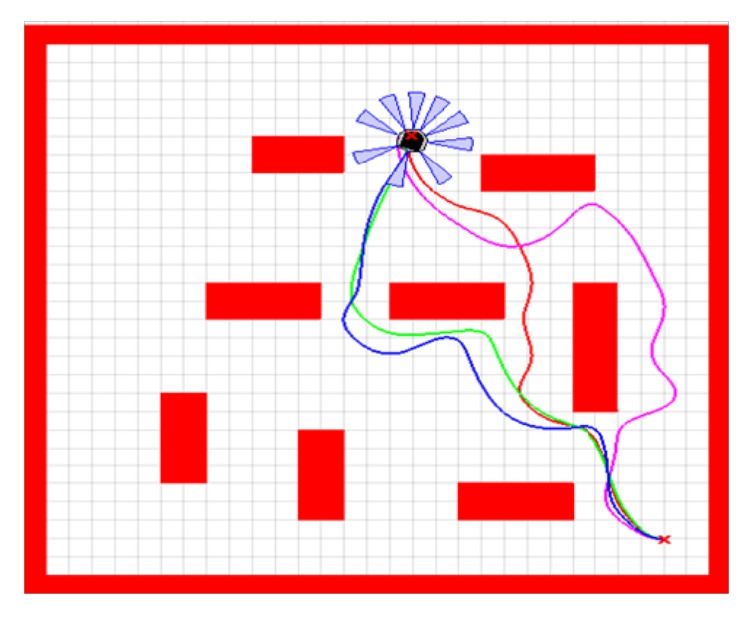
Motion of the mobile robot in the second strange environment.

Figures 11 and 12 depict the trajectory crossed by the mobile robot using the proposed method and other intelligent methods in two different strange environments. The red line is the path traced by the mobile robot using the TLBO-based ANFIS controller. The blue line is the trajectory followed by the robot using the IWO-based ANFIS controller. The green line is the trajectory of the robot using the BBO algorithm and the magenta one is the trajectory using the PSO algorithm.

To evince the efficiency and the power of the developed navigational controller, two significant criteria based on path length and travelling time are measured and given in Table 3. It can be clearly seen that TLBO-based ANFIS controller yields better results compared to other intelligent methods.

## 6. Conclusion

Four different evolutionary algorithms have been used to train an ANFIS controller for the navigation problem of mobile robot in a strange cluttered environment. These algorithms are PSO, IWO, BBO, and TLBO algorithms. Two main objectives are considered to minimize joint travelling time and total path length at the same time. Compared with the three other intelligent algorithms, TLBO-based ANFIS has performed very well for the studied navigational problem, while PSO algorithm performed poorly for the same problem. Real implementation of the announced approach will be developed in our future work. More intelligent evolutionary algorithms may be used.

## Figures and Tables

**Table 1:** Some training and testing dataset.

Angle_gtg_ao	Angular velocity
0.04575968951088669	−0.5136580646789393
0.05161782158495741	0.21358442837709696
0.05028751858455458	0.20346639427862612
0.04892471599559453	0.1967326390214124
0.04322374721979803	0.19129939110585853
0.035830613708575966	0.16764949463891513
0.043838843149825314	0.13775644295379605
0.039227218313333735	0.17289155273086265
0.03454703339172103	0.1519406961385048
0.029562255127593785	0.13322351795172563
0.024763088824827337	0.11323826735427753
0.02009879569488924	0.09409110607989632
0.015529357393452271	0.07547095759255704
0.011702662987861356	0.05721994003120612
0.008353106242134456	0.0420675625195056
0.005261204814970032	0.02876893962169187
0.002237266274856186	0.016455495579154156

**Table 2:** Comparison of performances of PSO-ANFIS, IWO-ANFIS, BBO-ANFIS, and TLBO-ANFIS models.

Optimization algorithms	Training data				Test data			
	MSE	RMSE	Mean error	Std. dev.	MSE	RMSE	Mean error	Std. dev.
PSO	0.11254	0.33547	0.0044081	0.33622	0.097484	0.31222	0.00022889	0.31392
IWO	0.018774	0.13702	0.0088525	0.13705	0.014758	0.12148	−0.016566	0.121
BBO	0.0038922	0.062388	0.0061697	0.062225	0.0072857	0.085536	0.00007763	0.085819
TLBO	0.00062319	0.024964	−0.0011524	0.024995	0.00066108	0.025691	−0.0028541	0.025691

**Table 3:** Comparison of simulation results between the TLBO-based ANFIS controller and other techniques.

Methods	Path length (m)		Travelling time (s)	
	Scenario 1	Scenario 2	Scenario 1	Scenario 2
PSO-based ANFIS	4.17	6.23	20.85	31.15
IWO-based ANFIS	3.61	5.46	18.05	27.3
BBO-based ANFIS	3.36	4.92	16.8	24.6
TLBO-based ANFIS	3,04	4.28	15.2	21.4
